# DNA Methylation Signatures of Systemic Inflammation Are Associated With Brain Volume, Cognitive Trajectories, and Long‐Term Dementia Risk

**DOI:** 10.1111/acel.70281

**Published:** 2025-11-22

**Authors:** Shannon M. Drouin, Perry Kuo, Cassandra Blew, Michael R. Duggan, Diefei Chen, Hannah M. Smith, Jingsha Chen, Katherine Giorgio, Gabriela Gomez, Ann Zenobia Moore, Guray Erus, Christos Davatzikos, Jan Bressler, Jeanette Simino, Yang Li, Rebecca F. Gottesman, Riccardo E. Marioni, Sanaz Sedaghat, Luigi Ferrucci, Susan M. Resnick, Keenan A. Walker

**Affiliations:** ^1^ Laboratory of Behavioral Neuroscience, National Institute on Aging Intramural Research Program Baltimore Maryland USA; ^2^ Translational Gerontology Branch, Intramural Research Program, National Institute on Aging NIH Baltimore Maryland USA; ^3^ Department of Epidemiology Johns Hopkins Bloomberg School of Public Health Baltimore Maryland USA; ^4^ Centre for Genomic and Experimental Medicine, Institute of Genetics and Cancer University of Edinburgh Edinburgh UK; ^5^ Division of Epidemiology & Community Health, School of Public Health University of Minnesota Minneapolis Minnesota USA; ^6^ Department of Medicine Brigham and Women's Hospital Boston Massachusetts USA; ^7^ Artificial Intelligence in Biomedical Imaging Laboratory, Perelman School of Medicine University of Pennsylvania Philadelphia Pennsylvania USA; ^8^ Department of Epidemiology, School of Public Health University of Texas Health Science Center at Houston Houston Texas USA; ^9^ Department of Data Science, John D. Bower School of Population Health University of Mississippi Medical Center Jackson Mississippi USA; ^10^ Department of Medicine NYU Grossman School of Medicine New York New York USA; ^11^ National Institute of Neurological Disorders and Stroke Intramural Research Program Bethesda Maryland USA

**Keywords:** biomarkers, cognitive dysfunction, dementia, epigenomics, inflammation, neuroimaging

## Abstract

C‐reactive protein (CRP) and growth differentiation factor 15 (GDF15) are important markers of inflammation associated with brain health. Compared to plasma, DNA methylation (DNAm) measures of CRP and GDF15 may provide stable epigenetic measures of chronic exposure to inflammation and could therefore be robustly predictive of inflammation‐related brain aging and neurodegeneration. We leveraged a subsample of Baltimore Longitudinal Study of Aging (BLSA) participants with DNAm/plasma data and longitudinal neuroimaging/cognition data (*n* = 430–1100). We used a proteome‐wide analysis to characterize the biology of DNAm CRP and GDF15, and latent growth curve models to explore the associations with longitudinal trajectories of 19 brain region volumes and five cognitive domains. Finally, we related DNAm/plasma CRP and GDF15 to dementia risk in two external cohorts. DNAm CRP and GDF15 showed a proteomic signature consistent with systemic immune activation. We identified several brain regions with significant associations between elevated DNAm CRP And GDF15 and (a) lower brain volume level (at age 75) and (b) greater rate of atrophy. Compared to plasma CRP, DNAm CRP was more strongly associated with brain volume, cognitive trajectories, and dementia risk. DNAm and plasma GDF15 were similarly associated with several total lobar, total lobar white matter, and AD‐relevant region trajectories and dementia risk, but DNAm measures outperformed plasma measures in relation to cognitive trajectories. Epigenetic signatures of CRP and GDF15 reflect immune and inflammation‐related pathway activation. These signatures, especially DNAm CRP, were associated with accelerated brain atrophy, cognitive decline, as well as long‐term dementia risk.

## Graphical abstract

The current study provides evidence that epigenetic proxies of CRP and GDF15 exposure reflect the activation of immune/inflammation‐related pathways and are associated with longitudinal brain atrophy and cognitive decline as well as long‐term dementia risk.

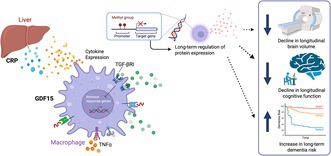

## 1 Background

Long‐term exposure to inflammation has been established as a key factor in brain and cognitive aging (Corlier et al. 2018; Franceschi and Campisi 2014; Walker et al. 2022; Walker, Le Page, et al. 2023). C‐reactive protein (CRP) is an inflammatory marker that has been consistently associated with adverse aging‐related morbidities. In older adults, increased levels of circulating CRP have been linked with higher rates of all‐cause morbidity and mortality (Hillary et al. 2024; Michaud et al. 2013), worse cognitive function and cognitive decline (Arce Rentería et al. 2020; Marsland et al. 2015; Singh‐Manoux et al. 2014; Tegeler et al. 2016), brain atrophy (Wang et al. 2022), and higher risk of Alzheimer's disease (AD) (Zhang et al. 2022). Similarly, growth differentiation factor 15 (GDF15)—also known as macrophage inhibitory cytokine 1 (MIC1)—has been identified as an anti‐inflammatory cytokine and a marker of immune‐mediated stress response that increases with age, and in the context of cellular senescence and certain age‐related diseases (e.g., cardiovascular disease and dementia) (Evans et al. 2024; Walker, Chen, et al. 2023).

Despite known links between inflammation and pathological aging outcomes, studies have found inconsistent associations between peripheral levels of inflammatory proteins, including CRP and GDF15, and markers of brain health and neurodegenerative disease (Dik et al. 2005; Ravaglia et al. 2007; Stevenson et al. 2020; Yang et al. 2015). A driver of these inconsistencies may be natural inter‐ and intra‐day fluctuations and variability in plasma protein levels as well as potential measurement error (Conole et al. 2021; Moldoveanu et al. 2000; Stevenson et al. 2021). Moreover, many health conditions, as well as physical and psychological stressors, can cause transient changes in inflammatory proteins that may further contribute to variability in inflammatory protein abundance. A consequence of this variability is a difficulty in accurately estimating an individual's long‐term exposure to inflammatory stimuli, i.e., chronic inflammation. Alternatively, chronic inflammation can be measured by quantifying epigenetic signatures (DNA‐methylation [DNAm]), which may act as determinants of inflammatory gene transcription (Gadd et al. 2022, 2024; Stevenson et al. 2021). Because DNAm, though modifiable, is more stable and less sensitive to inter‐ and intra‐day fluctuations than circulating protein levels and is trained on the inputs of protein‐DNAm associations from a large (13,399) number of individuals (Lu et al. 2022; Stevenson et al. 2020), blood‐based DNAm scores can be derived to estimate one's long‐term exposure to a given protein.

Previously, DNAm measures of GDF15 and CRP have been used as a component of composite variables (e.g., GrimAge version 2) to capture the immunologic contributions to accelerated biological aging, morbidity, and mortality. DNAm CRP has been associated with adverse neurocognitive outcomes (Conole et al. 2021; Smith et al. 2024). Specifically, Conole and colleagues found that DNAm CRP was significantly associated with cross‐sectional brain atrophy, white matter microstructure, and cognitive performance, and that epigenetic CRP measures were more strongly associated with measures of brain structure than were circulating CRP protein levels. Similarly, Smith et al. (2024) found that elevated DNAm CRP was cross‐sectionally associated with lower MRI‐defined brain volume, as well as greater dementia risk over a 16‐year follow‐up period (Smith et al. 2024). Although higher levels of GDF15 protein have also been linked to poor brain health and dementia risk (Isik et al. 2024; Walker et al. 2024; Walker, Chen, et al. 2023), less is known about the extent to which an epigenetic indicator of long‐term GDF15 exposure (DNAm GDF15) relates to adverse neurocognitive outcomes (Gadd et al. 2024).

The current study examined DNAm measures of CRP and GDF15—two inflammatory proteins with distinct immunologic significance—and extended previous cross‐sectional findings using longitudinal MRI imaging and cognitive data in a large cohort of Baltimore Longitudinal Study of Aging (BLSA) adults. We conducted a proteome‐wide analysis to identify functional pathways associated with blood DNAm CRP and DNAm GDF15 scores and examined how each of these putative markers of chronic inflammation was associated with longitudinal measures of brain structure as well as cognitive function among older adults. Additionally, we compared the performance of the CRP and GDF15 DNAm scores to that of their plasma protein counterparts and determined whether the DNAm associations extended to near‐ and long‐term dementia risk in two independent cohort studies.

## 2 Methods

### 2.1 Participants and Study Design

**FIGURE 1:**
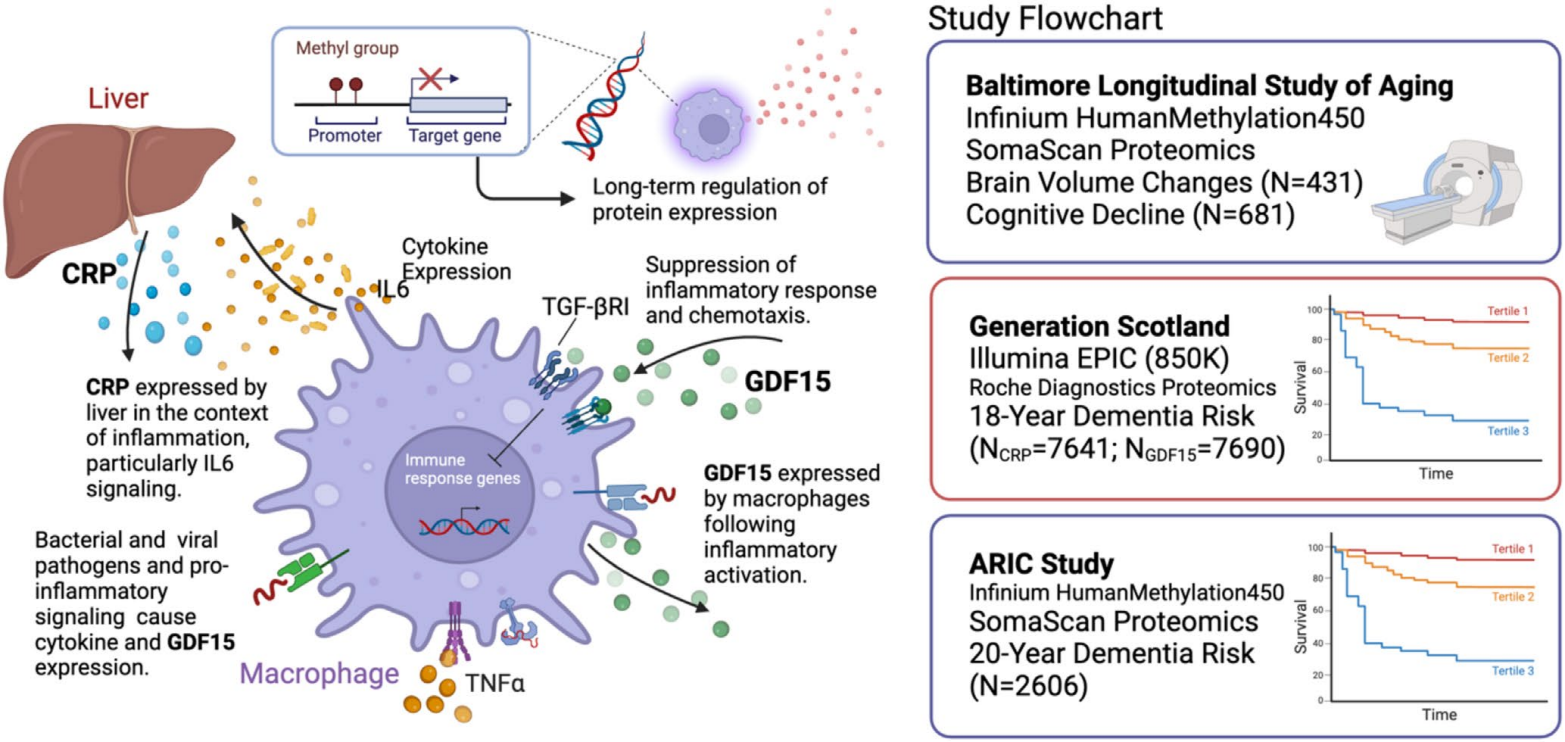
Study design.

Participants for this study were selected from the BLSA. The BLSA is an ongoing cohort study of human aging that started in 1958, the details of which have been extensively reported elsewhere (Shock 1984). BLSA protocols were approved by the National Institutes of Health Intramural Research Program Institutional Review Board (Protocol number 03‐AG‐0325). Informed consent was obtained from all participants. The study analysis plan and sample selection are presented in Figure 1, Table S1, and Figure S1. For our proteomic characterization of DNAm scores, we selected participants with DNAm and plasma protein measurements at the same visit (*n* = 186). For analyses relating DNAm to brain volume trajectories, we selected participants with available DNAm data (*n* = 768) who also had at least one and up to eight MRI measurements concurrently and/or after plasma collection (*n* = 430). For analyses relating DNAm to cognitive trajectories, we selected participants with available DNAm data who also had at least one and up to eight cognitive assessments concurrently and/or after plasma collection (*n* = 680). For our analyses comparing the strength of DNAm associations to that of plasma protein levels, we selected participants with available plasma protein data closest to the available DNAm visit (if available) for both the MRI outcomes (*n* = 1076) and the cognitive outcomes (*n* = 1100).

### 2.2 DNA Methylation and Plasma Protein Measurement

DNAm was measured in the BLSA using the Illumina Infinium HumanMethylation450 BeadChip (Illumina Inc., San Diego, CA). DNA samples from whole blood were collected between November 1993 and March 2010 and processed consistent with manufacturer instructions; background correction, normalization, and quality control of data were conducted using the minfi package (28, 30). DNAm measures of CRP (132 CpG sites) and GDF15 (137 CpG sites) were estimated from CpG methylation status of 485,577 CpG sites (as per GrimAgeV2 calculations). Missing measures were median‐imputed. As was done in a previous study, elastic net regression models were used in R (glmnet) to regress each plasma protein on CpGs (Lu et al. 2019). CpG sites and coefficients for DNAm CRP and GDF15 are publicly available (https://github.com/bio‐learn/biolearn/blob/master/biolearn/data/GrimAgeV2.csv). DNAm GDF15 and CRP are calculated by a linear combination of each coefficient with the methylation level at each corresponding CpG site. DNAm CRP and DNAm GDF15 were standardized (*z*‐score) for all analyses. As such, all results should be interpreted as the raw change in neuroimaging and cognitive factor score per standard deviation increase in DNAm CRP or GDF15. We additionally used DNAm data to calculate immune cell proportion (Horvath 2013) to be included as a covariate in subsequent analyses.

Plasma CRP and GDF15 were measured using the SomaScan Assay v4.1 (7289 total SOMAmer reagents), as has been described previously (Candia et al. 2022). After SomaLogic and BLSA quality control procedures, plasma CRP and GDF15 values were log_2_ transformed and winsorized at five standard deviations. Importantly, the SomaScan measures of CRP and GDF15 have been previously validated in multiple studies, including by our group (Rooney et al. 2023; Tin et al. 2019; Walker, Chen, et al. 2023). The intra‐assay coefficient of variation was 7.0% and 9.5% for CRP and GDF15, respectively, as defined by blind duplicates measured in the BLSA (a subsample of the current analytic sample). Plasma CRP and GDF15 were standardized (*z*‐score) for all analyses.

### 2.3 MRI/Imaging Data

A subset of participants in the BLSA were included in the BLSA Neuroimaging Substudy. These participants completed T1‐weighted magnetization‐prepared rapid gradient echo (MPRAGE) scans (TR = 6.8 ms, TE = 3.2 ms, flip angle = 8°, image matrix = 256 × 256 × 170, voxel size = 1.0 × 1.0 × 1.2 mm^3^, sagittal acquisition) on a 3 T (Philips Achieva). Volumes of anatomical regions of interest were segmented using Multi‐atlas Region Segmentation Utilizing Ensembles (Doshi et al. 2016). Primary neuroimaging outcomes included total brain, total gray matter, total white matter, and ventricular CSF volume, as well as two machine‐learning derived multidimensional brain atrophy patterns: Spatial Patterns of Abnormality for Recognition of Early AD (SPARE‐AD) and SPARE‐Brain Aging (SPARE‐BA; a brain age index) (Davatzikos et al. 2009; Habes et al. 2016). Briefly, higher SPARE‐AD scores indicate a participant has MRI‐defined structural brain features more like that of individuals with AD. SPARE‐BA uses MRI‐defined structural brain features to estimate an individual's brain age. As part of our secondary analyses, we examined several additional regional brain volumes: total lobar (frontal, temporal, parietal, and occipital lobes), total lobar white matter (frontal, parietal, temporal, and occipital white matter) and AD‐relevant regions (hippocampus, middle temporal gyrus, angular gyrus, amygdala, middle cingulate gyrus) (Dickerson et al. 2011; Graff‐Radford et al. 2017).

### 2.4 Cognitive Data

Included cognitive domains were general cognition, executive function, memory/semantic retrieval, and visuospatial functioning. To assess memory/semantic retrieval, we used test results from the California verbal learning free recall including both short and long delay trials, Boston naming, letter fluency, and category fluency. These indicators represent a broad latent construct which taps into episodic memory, semantic memory, and semantic retrieval. For executive function/attention, we used digits forwards and backwards, digit symbol substitution, trail making Part A and Part B, and similarities. For visuospatial functioning, we used card rotation, clock drawing, and Benton errors. Using all the above mentioned items, we fitted a unidimensional Confirmatory Factor Analysis model to derive the general cognition factor score for each BLSA participant with at least one non‐missing cognitive test at each visit and who was aged > 60 years old at the baseline visit. We additionally allowed residual correlations between items within each cognitive domain (RMSEA = 0.047, CFI = 0.956, TLI = 0.923, SRMR = 0.038; Table S2). Secondly, we fitted a three correlated‐factor Confirmatory Factor Analysis model to derive factor scores for each memory/semantic retrieval, executive function, and visuospatial functioning domain. We introduced residual correlations among different trials from the same test (RMSEA = 0.048, CFI = 0.937, TLI = 0.920, SRMR = 0.047; Table S2). All the above analyses were carried out using MPlus Version 8.2.

### 2.5 Alzheimer's Disease and Related Dementia (ADRD) Biomarkers

Plasma ADRD biomarkers were measured concurrently with epigenetic data collection (*n* = 67–159) and plasma CRP and GDF15 quantification (*n* = 898–1410) for a subset of BLSA participants. Single Molecule Array (Simoa) Neurology 4‐Plex E (N4PE) and pTau‐181 (v.2) assays on the Simoa HD‐X instrument (Quanterix) were used to measure Aβ_40_, Aβ_42_, GFAP, NfL, and pTau‐181 plasma concentrations. All assays were run in duplicate and values averaged. CVs were 2.8%, 1.9%, 5.0%, 5.1%, and 4.4% for Aβ_40_, Aβ_42_, GFAP, NfL, and pTau‐181, respectively. We used the Aβ_42/40_ ratio in all analyses.

### 2.6 Dementia Risk in the Generation Scotland Study

The Generation Scotland: Scottish Family Health Study (GS) is a large cohort study (> 20,000 participants) featuring genetic, epigenetic, clinical, lifestyle, and sociodemographic data. Recruited GS participants were between the ages of 17 and 99 years at study baseline (*M* = 47.5 [14.93]; 58.8% female) between 2006 and 2011. The GS sample has been described in detail previously (Smith et al. 2013). Dementia diagnosis was determined using electronic health records data linkage (Smith et al. 2024). Incident dementia diagnoses between GS baseline visit (2006–2011) and the last date of electronic health record linkage (October 2023) were used for the current analyses. An age filter of ≥ 65 years was applied to age at diagnosis to account for early onset dementia, and age at censor (October 2023) to ensure the control group was age appropriate. GS DNAm methods have been described in detail elsewhere (Gadd et al. 2024; Smith et al. 2024). Using whole blood obtained at the baseline visit (2006–2011), DNAm was analyzed using the Illumina EPIC array (850K) at > 850,000 CpG sites for 18,869 GS samples (Milbourn et al. 2024). CRP and GDF15 plasma protein levels were measured from blood collected at the baseline visit using cobas c311 (Roche Diagnostics, UK) and e411 (Roche Diagnostics, Basel, Switzerland) analyzers, respectively (Gadd et al. 2024; Hillary et al. 2024).

### 2.7 Dementia Risk in the Atherosclerosis Risk in Communities Study

The Atherosclerosis Risk in Communities (ARIC) study is a prospective epidemiologic study conducted in four US communities (Forsyth County, NC; Jackson, MS; the northwest suburbs of Minneapolis, MN; and Washington County, MD), which enrolled 15,792 mostly white and black participants aged 45–64 between 1987 and 1989. A surveillance approach was used for dementia diagnosis, which was based on cognitive assessments, telephone screening, informant ratings, hospital records, and death record review. Dementia was classified based on algorithm and adjudication by a group of experts based on the NIA/AA (National Institute on Aging and Alzheimer's Association) and Diagnostic and Statistical Manual of Mental Disorder—Fifth Edition (DSM‐V) criteria. Incident dementia diagnoses between ARIC visit 2 (1990–1993) and visit 7 (2018–2019) were included in the current analyses (Knopman et al. 2024). DNAm in ARIC was measured at visit 2 using the Illumina Infinium HumanMethylation450 (HM450) BeadChip which surveyed 480,000 cytosine‐guanine (CpG) dinucleotide sites (Bose et al. 2014; Hahn et al. 2023) from whole blood. CRP and GDF15 protein levels were measured using the SomaScan Assay v4.0 at visit 2; SomaLogic protein quality control steps in ARIC have been described in detail previously (Walker et al. 2021).

### 2.8 Statistical Analyses

#### 2.8.1 Relating DNAm CRP and DNAm GDF15 to Brain Volume Trajectories

We conducted a series of conditional latent growth models in Mplus 8.2. For each of the 19 brain region outcomes of interest, we tested two separate prediction models: (1) using DNAm CRP and (2) using DNAm GDF15. In each model, we included chronological age as the metric of change (centered at age 75) as well as intracranial volume at age 70 (time‐invariant) and scanner type (time‐varying) as covariates. Intracranial volume was centered at the sample mean. We report FDR‐corrected *p* values across brain regions for each parameter of interest (intercept for CRP, slope for CRP, intercept for GDF15, slope for GDF15).

Following our initial analyses in the entire sample, we stratified each conditional latent growth model (limited to six visits due to small male/female group sizes at later follow‐up periods) for the primary imaging outcomes by sex to identify sex‐specific predictor effects of DNAm CRP and GDF15. To test whether stratification improved model fit, we compared the unconstrained stratified model with a constrained stratified model where the effect of the predictor of interest (DNAm CRP or GDF15) on intercept and slope was fixed to be the same across males and females. We used the difference in the −2 loglikelihood (−2LL) values between these two models and the difference in the degrees of freedom. A significant difference value indicated significant sex moderation.

#### 2.8.2 Relating DNAm CRP and DNAm GDF15 to Cognitive Trajectories

We used conditional latent growth models in Mplus 8.2 to test the prediction of general cognitive, memory/semantic retrieval, executive function, and visuospatial processing trajectories by DNAm CRP and DNAm GDF15. For all models, we included age as the metric of change as well as the following covariates: sex (time‐invariant) and years of education (time‐invariant). Education was centered at the mean. DNAm CRP and DNAm GDF15 were standardized. Following our initial analyses in the entire sample, we stratified each conditional latent growth model (limited to six visits) by sex and evaluated sex moderation effects as described above.

#### 2.8.3 Comparing Plasma and DNAm Measures of CRP and GDF15

We ran a series of parallel conditional latent growth models for all outcomes of interest using plasma measures of CRP and GDF15 in place of the DNAm measure. These models included the same covariates described above but were conducted in a larger sample, as a larger subsample of BLSA participants had available plasma data (compared to DNAm). As we did for the DNAm analyses, we report FDR‐corrected *p* values across brain regions for each parameter of interest (intercept for CRP, slope for CRP, intercept for GDF15, slope for GDF15).

#### 2.8.4 Relating DNAm and Plasma CRP and GDF15 to Dementia Risk

To determine whether DNAm CRP and GDF15 (as compared to plasma counterparts) are associated with dementia risk, we used data from two external cohorts: Generation Scotland (*n*_crp_ = 7641, *n*_gdf15_ = 7690) and the Atherosclerosis Risk in Communities (ARIC) study (*n* = 2606). Demographic and baseline information for the validation cohorts can be found in Table S1.

In the Generation Scotland (R 4.41) and ARIC cohorts (R 4.1.3), we used Cox proportional hazard models (*survival*, *coxPH*) to test associations between time‐to‐dementia and GDF15/CRP (DNAm scores and plasma levels). Three models were used with each predictor of interest separately (DNAm CRP, plasma CRP, DNAm GDF15, plasma GDF15): (a) Model 0 (unadjusted), (b) Model 1 (adjusted for age and sex), and (c) Model 2 (adjusted for age, sex, BMI, education qualification, diabetes, high blood pressure, and smoking). In the Generation Scotland cohort only, Cox mixed effects models (R 4.41, *coxme* 2.2.22) were also used in parallel to account for relatedness as has been done previously (Smith et al. 2024).

## 3 Results

### 3.1 Biological Characterization of DNAm CRP and DNAm GDF15

**FIGURE 2:**
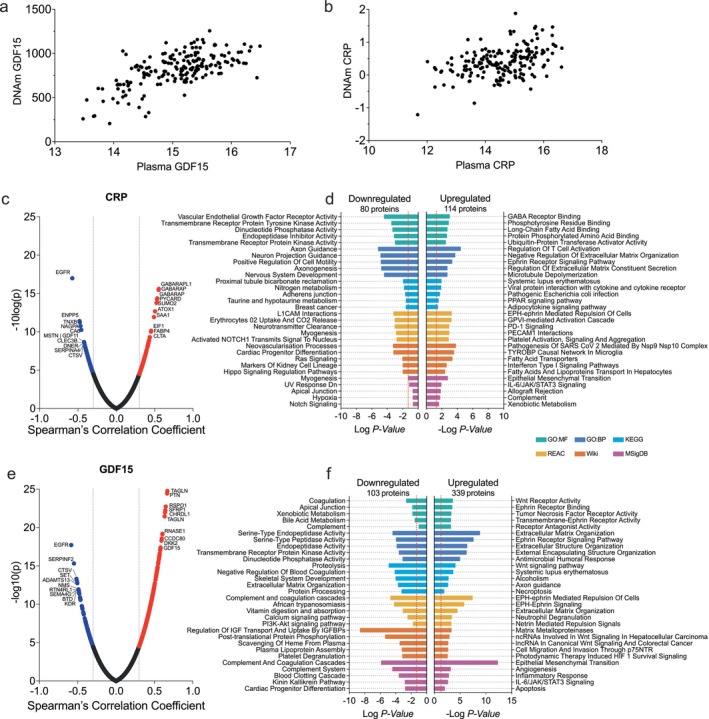
Association between DNAm GDF15 and CRP and the plasma proteome in participants with concurrent samples (*n* = 186). Panels a and b show the correlation between plasma and DNAm GDF15 (a) and CRP (b). Panel c displays the volcano plot of correlations between DNAm CRP and the plasma proteome (SomaScan v4.1) in the BLSA for participants with concurrent measurements (*N* = 186). Panel d shows the enrichment of plasma proteins which showed the strongest negative (left) and positive (right) correlations with DNAm CRP. Panel e displays the volcano plot of correlations between DNAm GDF15 and the plasma proteome (SomaScan v4.1) in the BLSA for participants with concurrent measurements (*N* = 186). Panel f shows the enrichment of plasma proteins which showed the strongest negative (left) and positive (right) correlations with DNAm GDF15.

In a subset of 186 BLSA participants (*M* age: 74.68 [12.98 SD], 47.3% female) with concurrently measured protein levels and DNAm, we found that both DNAm CRP (*r*_s_ = 0.41) and DNAm GDF15 (*r*_s_ = 0.60) were strongly correlated with their plasma counterparts (Figure 2a,b). Correlations between DNAm and plasma measures were weaker, but still moderate, in the Generation Scotland (*r*_s CRP_ = 0.34; *r*_s GDF15_ = 0.35) and ARIC (*r*_s CRP_ = 0.37; *r*_s GDF15_ = 0.44) cohorts. In all participants with eligible epigenetic data in the BLSA, DNAm CRP and DNAm GDF15 were strongly correlated (*r*_s_ = 0.44, *p* < 0.005). Similarly, in all participants with available plasma data in the BLSA, plasma CRP and plasma GDF15 were comparatively weakly correlated (*r*_s_ = 0.10, *p* < 0.005).

To determine whether DNAm CRP and DNAm GDF15 were indeed proxies for inflammation, we used a series of pathway analyses. Specifically, we used a correlation threshold of *r*_s_ > 0.3 to identify which of the 7000 plasma proteins (as measured by SomaScan Assay v4.1) were associated with each DNAm (Tables S3 and S4; Figure 2). The identified protein set was then submitted as inputs in overrepresentation analyses conducted using Enrichr (Xie et al. 2021) and Ingenuity Pathway Analyses (IPA) (Krämer et al. 2014). Using an absolute value correlation threshold of 0.3 for over‐representation analyses (194 proteins, *p*‐value of approximately *p* < 3.0E‐5; Figure 2c; Table S5), proteins positively associated with DNAm CRP were enriched for pro‐inflammatory pathways, including inflammatory response, cytokine and chemokine activity (e.g., IL‐6/JAK/STAT3 signaling), and immune‐mediated disease pathways (Figure 2d; Table S4).

Using a similar correlation threshold (442 proteins; Figure 2e; Table S6), over‐representation analyses identified several immune‐related pathways among plasma proteins both positively and negatively associated with DNAm GDF15, including immune‐mediated disease pathways, neutrophil degranulation, and complement signaling (Table S6; Figure 2f). However, the DNAm GDF15 proteomic signature was also strongly enriched for ephrin receptor activity and extracellular matrix organization. These results were supported by canonical pathway analyses conducted using Ingenuity Pathway Analysis (IPA; Table S6).

### 3.2 DNAm CRP and DNAm GDF15 Are Associated With Brain Volume Trajectories

**FIGURE 3:**
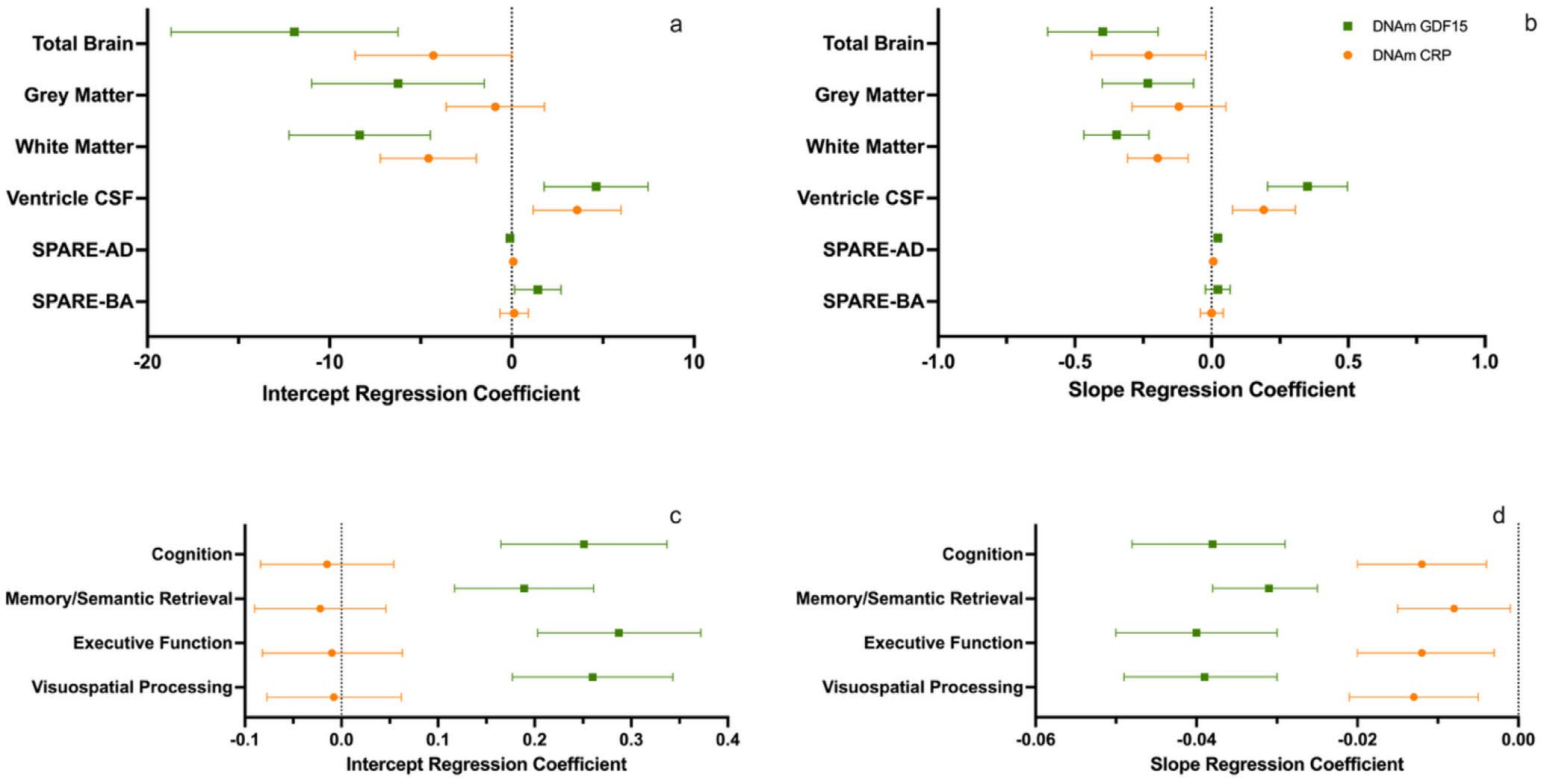
DNAm CRP and GDF15 intercept and slope regression coefficients for measures of brain volume and cognition. Panels a and b compare the intercept (a) and slope (b) regression coefficients (95% CIs) for the primary brain regions of interest. Panels c and d compare the intercept (c) and slope (d) regression coefficients (95% CIs) for the four cognitive trajectories.

**FIGURE 4:**
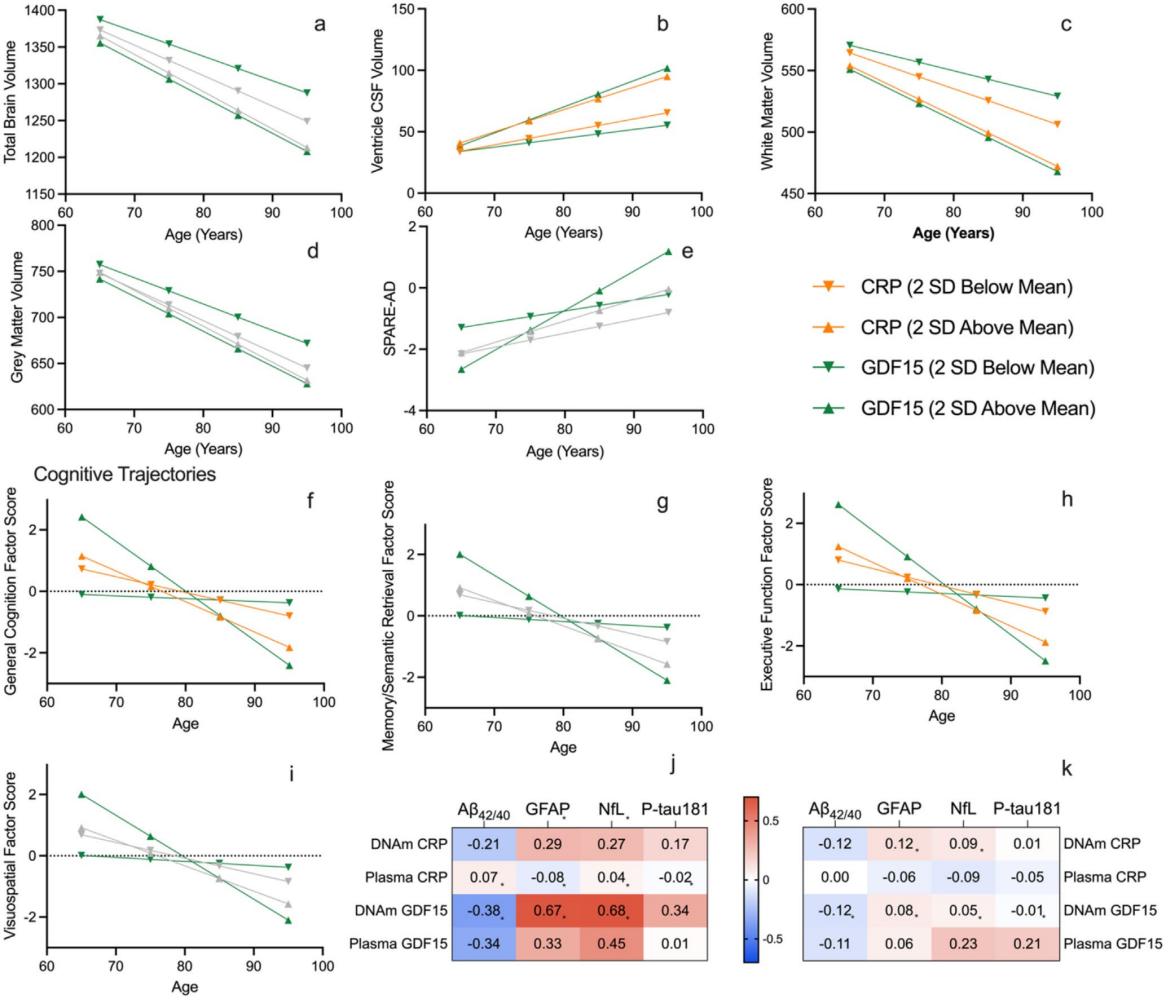
Estimated brain volume and cognitive trajectories as predicted by DNAm CRP and GDF15. Estimated trajectory plots for primary imaging (a–e) and cognitive outcomes (f–i) which demonstrated a significant effect of GDF15 or CRP on change over time (slope). Green lines show the effect of GDF15 on level at age 75 (intercept) and change over time (slope). Orange lines indicate the effect of CRP on level at age 75 (intercept) and change over time (slope). Gray lines indicate a nonsignificant effect of GDF15 and/or CRP on change over time (slope) after FDR correction. Panel J (unadjusted) and K (partial correlation with age) show the Spearman correlation coefficients between DNAm CRP/GDF15 and Aβ40/42 ratio (*n* = 159)/GFAP (*n* = 159)/NFL (*n* = 158)/P‐tau181 (*n* = 67) as well as plasma CRP/GDF15 and Aβ40/42 ratio (*n* = 1409)/GFAP (*n* = 1410)/NFL (*n* = 1409)/P‐tau181 (*n* = 898). Asterisks (*) indicate a significant Spearman correlation coefficient at *p* < 0.05.

In a sample of 430 BLSA adults (*M* age: 69.8 [12.1 SD], 54.1% female) with longitudinal imaging data, DNAm CRP and DNAm GDF15 scores were associated with level (at age 75) in volume and changes over time (slope) in several brain regions following FDR correction (Table 1 and Table S7, Figures 3 and 4). Among primary brain regions, higher levels of DNAm CRP and GDF15 were significantly associated with lower white matter volume at age 75 (*β*_CRP_ = −4.58 [1.38, *p* = 0.006], *β*_GDF15_ = −8.35 [1.98, *p* < 0.001]), higher ventricle CSF volume at age 75 (*β*_CRP_ = 3.58 [1.2, *p* = 0.02], *β*_GDF15_ = 4.62 [1.46, *p* = 0.004]), declines in total white matter volume (*β*_CRP_ = −0.20 [0.05, *p* < 0.001], *β*_GDF15_ = −0.34 [0.06, *p* < 0.001]), and increases in ventricle CSF volume (*β*_CRP_ = 0.19 [0.06, *p* = 0.009], *β*_GDF15_ = 0.35 [0.06, *p* < 0.001]). In addition, elevated DNAm GDF15 was uniquely associated with lower levels of nearly all primary brain regions and greater rates of atrophy in three primary regions following FDR correction, including steeper total brain (*β*_GDF15_ = −0.40 [0.10, *p* < 0.001]) and gray matter (*β*_GDF15_ = −0.23 [0.09, *p* = 0.007]) declines, as well as an increase in SPARE‐AD (*β*_GDF15_ = 0.02 [0.004, *p* < 0.001]) score. Across all significant associations, the effect of DNAm GDF15 on both intercept and slope appeared to be uniformly larger in magnitude than that of DNAm CRP (Figure 3).

**TABLE 1:** Regression coefficients derived from the conditional latent growth models relating DNAm and plasma CRP and GDF15 to primary brain volume regions.

Brain region		CRP: intercept coefficient [SE]	CRP: slope coefficient [SE]	GDF15: intercept coefficient [SE]	GDF15: slope coefficient [SE]
Primary imaging outcomes: DNAm	Total brain	−4.303* [2.194]	−0.23* [0.106]	−11.933** [3.455]	−0.398** [0.103]
	Gray matter	−0.906 [1.375]	−0.120 [0.087]	−6.25** [2.416]	−0.233** [0.085]
	White matter	−4.579** [1.345]	−0.197** [0.057]	−8.351** [1.975]	−0.348** [0.06]
	SPARE‐AD	0.07 [0.052]	0.006 [0.003]	−0.11 [0.077]	0.023** [0.004]
	SPARE‐BA	0.124 [0.399]	< 0.005 [0.021]	1.422** [0.647]	0.023 [0.023]
	Ventricle CSF	3.580** [1.227]	0.191** [0.058]	4.620** [1.458]	0.351** [0.057]
Primary imaging outcomes: Plasma	Total brain	−0.188 [1.301]	−0.097 [0.066]	−9.257** [1.513]	−0.458** [0.065]
	Gray matter	−0.071 [0.837]	−0.022 [0.047]	−5.454** [1.030]	−0.248** [0.048]
	White matter	−0.202 [0.843]	−0.072 [0.04]	−6.230** [1.009]	−0.408** [0.040]
	SPARE‐AD	< 0.005 [0.03]	−0.002 [0.002]	0.173** [0.038]	0.014** [0.002]
	SPARE‐BA	0.012 [0.257]	0.004 [0.014]	1.16** [0.307]	0.046** [0.014]
	Ventricle CSF	1.176 [0.687]	0.047 [0.033]	4.061** [0.767]	0.306** [0.037]

*Note:* Models included intracranial volume at age 70 and scanner type as covariates.

* *p* < 0.05.

** *p* < 0.05 after FDR correction.

The effect of DNAm CRP on primary brain volume trajectories was significantly moderated by sex for white matter volume (Δ‐2LL = 6.46, Δdf = 2) and ventricle CSF volume (Δ‐2LL = 74.61, Δdf = 2; Table S8, Figure S2). For DNAm GDF15, significant sex‐based moderation effects were found for total brain (Δ‐2LL = 25.87, Δdf = 2), total white matter (Δ‐2LL = 10.53, Δdf = 2), and SPARE‐AD (Δ‐2LL = 13.02, Δdf = 2). Across these regions, predictor effects on both intercept and slope were larger in magnitude in males compared to females.

### 3.3 DNAm CRP and DNAm GDF15 Are Associated With Cognitive Trajectories

In a sample of 680 BLSA adults (*M* age: 73.1 [9.5 SD], 48.7% female) with longitudinal cognitive data, a higher DNAm CRP score was not associated with the level for any of the cognitive outcomes at age 75, but was associated with longitudinal declines in general cognition (*β*_s_ = −0.01 [0.004, *p* = 0.003]), memory/semantic retrieval (*β*_s_ = −0.01 [0.004, *p* = 0.026]), executive function (*β*_s_ = −0.01 [0.004, *p* = 0.007]) and visuospatial processing (*β*_s_ = −0.01 [0.004, *p* = 0.002]; Figures 3 and 4). Elevated DNAm GDF15 was associated with both a higher level (at age 75) and faster declines in all four tested cognitive outcomes (Table 2, Figures 3 and 4). This included general cognition (*β*_i_ = 0.25 [0.04, *p* < 0.001]; *β*_s_ = −0.04 [0.005, *p* < 0.001]), memory/semantic retrieval (*β*_i_ = 0.19 [0.03, *p* < 0.001]; *β*_s_ = −0.03 [0.003, *p* < 0.001]), executive function (*β*_i_ = 0.29 [0.04, *p* < 0.001]; *β*_s_ = −0.04 [0.005, *p* < 0.001]), and visuospatial processing (*β*_i_ = 0.26 [0.04, *p* < 0.001]; *β*_s_ = −0.04 [0.005, *p* < 0.001]). Stratified models revealed no significant moderation effects of sex for either predictor (Table S8). Across brain and cognitive trajectory outcomes, our results were generally similar when we ran a sensitivity analysis controlling for estimated cell proportion (Table S9).

**TABLE 2:** Regression coefficients from the conditional latent growth models relating DNAm and plasma CRP and GDF15 to cognition.

		CRP: intercept coefficient [SE]	CRP: slope coefficient [SE]	GDF15: intercept coefficient [SE]	GDF15: slope coefficient [SE]
Predictor: DNAm	General cognition	−0.015 [0.035]	−0.012* [0.004]	0.251* [0.044]	−0.038* [0.005]
	Memory/semantic retrieval	−0.022 [0.035]	−0.008* [0.004]	0.189* [0.037]	−0.031* [0.003]
	Executive function	−0.01 [0.037]	−0.012* [0.004]	0.287* [0.043]	−0.040* [0.005]
	Visuospatial processing	−0.008 [0.035]	−0.013* [0.004]	0.260* [0.042]	−0.039* [0.005]
Predictor: Plasma	General cognition	−0.065* [0.029]	0.003 [0.003]	0.101* [0.03]	−0.016* [0.004]
	Memory/semantic retrieval	−0.039 [0.025]	0.002 [0.002]	0.073* [0.027]	−0.013* [0.003]
	Executive function	−0.055* [0.027]	0.002 [0.003]	0.098* [0.029]	−0.016* [0.004]
	Visuospatial processing	−0.061* [0.029]	0.003 [0.003]	0.098* [0.029]	−0.016* [0.004]

*Note:* Models included sex and education (in years) as covariates.

* *p* < 0.05.

### 3.4 DNAm CRP, DNAm GDF15, and ADRD Biomarkers

Higher DNAm CRP was significantly associated with a lower Aβ_40/42_ ratio (*r*_s_ = −0.21, *p* = 0.008), and greater GFAP (*r*_s_ = 0.29, *p* < 0.001) and NfL (*r*_s_ = 0.27, *p* < 0.001), but not with ptau‐181 (r_s_ = 0.16, *p* = 0.18) in participants with concurrent measures (Figure 4j; Table S10). Higher DNAm GDF15 was significantly associated with a lower Aβ_40/42_ ratio (*r*_s_ = −0.38, *p* < 0.001) and higher ptau‐181 (*r*_s_ = 0.34, *p* = 0.005), and showed especially strong correlations with greater levels of GFAP (r_s_ = 0.67, *p* < 0.001) and NfL (*r*_s_ = 0.68, *p* < 0.001). However, after adjusting for age, correlations between DNAm CRP, DNAm GDF15, and each of these plasma ADRD biomarkers were substantially attenuated and nonsignificant (Figure 4k; Table S10).

### 3.5 Comparing Effect Estimates From DNAm CRP and DNAm GDF15 Models to Estimates From Models Incorporating Plasma CRP and Plasma GDF15

**FIGURE 5:**
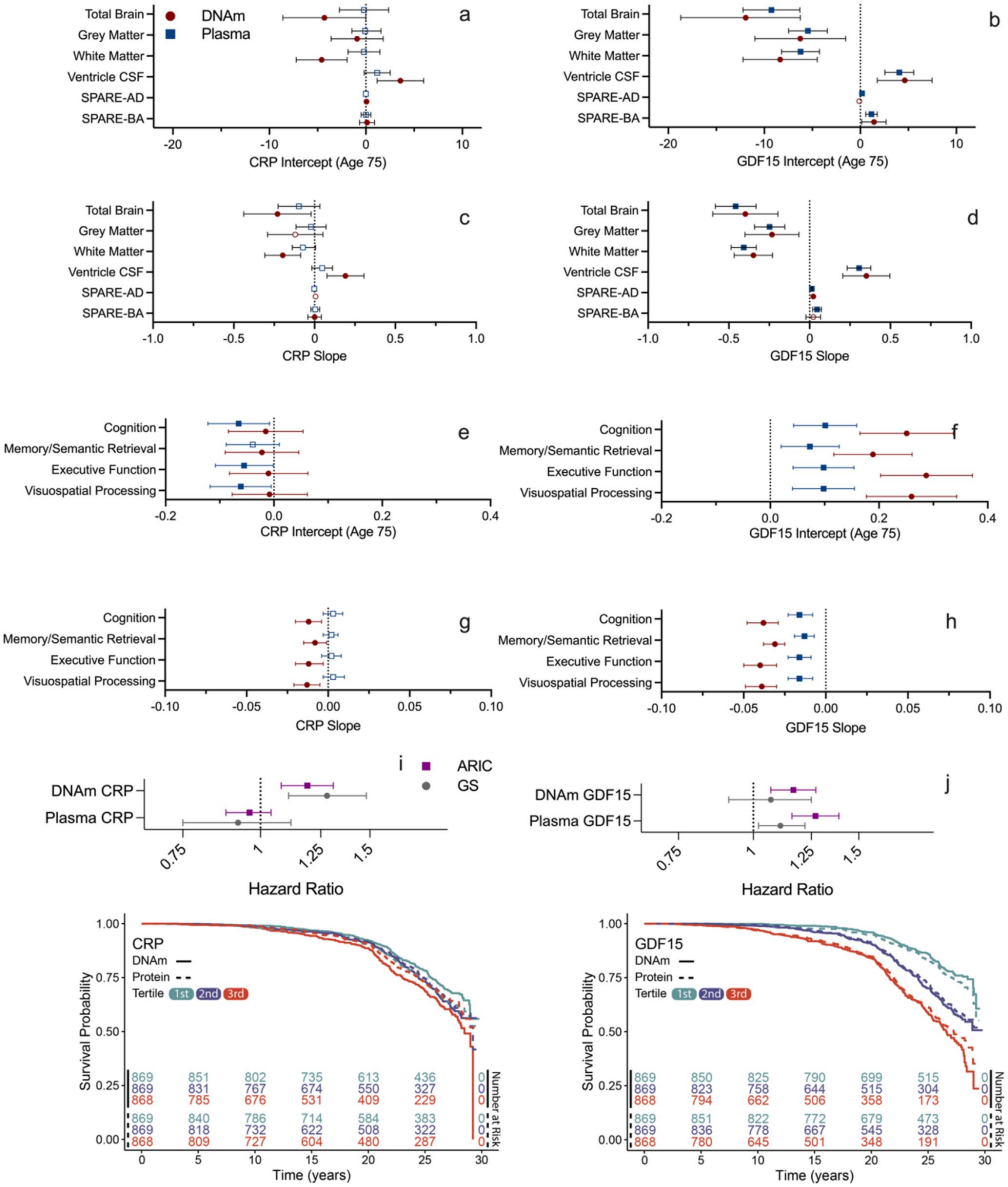
Comparison of DNAm versus plasma regression coefficients relating CRP and GDF15 to brain volume and cognitive trajectories. Panels a and b show the intercept (a) and slope (b) regression coefficients for plasma and DNAm CRP for the six primary brain trajectory outcomes. Panels c and d show the intercept (c) and slope (d) regression coefficients for plasma and DNAm for CRP for the cognitive trajectories. Panels e and f show the intercept (e) and slope (f) regression coefficients for plasma and DNAm GDF15 for the primary brain trajectories. Panels g and h show the intercept (g) and slope (h) regression coefficients for plasma and DNAm for GDF15 for the cognitive trajectories. Unfilled shapes indicate a nonsignificant regression coefficient.

In a sample of BLSA participants with plasma measures of CRP and GDF15, as well as 3T MRI (*n* = 1076; *M* age: 66.5 [14.9 SD]; 53.9% female) and cognitive measures (*n* = 1100; *M* age: 74.3 [8.7 SD]; 52.1% female), plasma CRP was not associated with primary brain volume level or change over time (Table 1). However, higher plasma CRP was associated with a lower level (but not declines) of general cognition (*β*_i_ = −0.065 [0.03, *p* = 0.026]), executive function (*β*_i_ = −0.055 [0.03, *p* = 0.045]), and visuospatial processing (*β*_i_ = −0.061 [0.03, *p* = 0.033]) at age 75 (Table 2). Overall, the effect of DNAm CRP appeared uniformly larger across all tested brain regions and cognitive domains as compared to plasma CRP (Figure 5a–d).

Greater plasma GDF15 was associated with lower total brain (*β*_i_ = −9.257 [1.51, *p* < 0.001]), gray matter (*β*_i_ = −5.454 [1.03, *p* < 0.001]), white matter (*β*_i_ = −6.230 [1.01, *p* < 0.001]), and higher ventricle CSF volumes (*β*_i_ = 4.06 [0.77, *p* < 0.001]), SPARE‐AD (*β*_i_ = 0.173 [0.04, *p* < 0.001]), and SPARE‐BA (*β*_i_ = 1.16 [0.31, *p* < 0.001]) at age 75. In addition, greater plasma GDF15 was associated with declines in total brain (*β*_s_ = −0.458 [0.07, *p* < 0.001]), gray matter (*β*_s_ = −0.248 [0.05, *p* < 0.001]), and white matter (*β*_s_ = −0.408 [0.04, *p* < 0.001]) volumes and increases in SPARE‐AD (*β*_s_ = 0.014 [0.002, *p* < 0.001]), SPARE‐BA (*β*_s_ = 0.046 [0.014, *p* = 0.001]) and CSF ventricle volume (*β*_s_ = 0.306 [0.037, *p* < 0.001]; Table 1). Like DNAm GDF15, plasma GDF15 was associated with better cognition at age 75 and greater rates of cognitive decline in all cognitive domains (Table 2). The effect for DNAm GDF15 appeared larger than that of plasma GDF15 for brain volumes as well as cognition level and slopes. On the other hand, DNAm GDF15 appeared to have smaller effects than plasma GDF15 on brain volume slopes. Across outcomes, our results were similar when we restricted the plasma analyses to the approximate subgroup of participants with epigenetic data only (Table S9).

### 3.6 DNAm CRP and DNAm GDF15 Scores Are Associated With Dementia Risk

To determine whether the DNAm associations extended to dementia risk among older adults, we examined participants from the GS study (*n* = 7641; *M* age: 60.5 [7.2 SD]; 58.1% female) with a maximum of 18 years of dementia follow‐up (median follow‐up = 13.8 years; median time‐to‐event = 9.17 years; 206 dementia cases). A one standard deviation (SD) increase in DNAm CRP was associated with a 28% increase [HR: 1.28; 95% CI: 1.11–1.48] in dementia risk after adjusting for age and sex (*p* = 0.0007; Figure 5). On the other hand, dementia risk was unrelated to plasma CRP (HR: 0.92; 95% CI: 0.75–1.12; *p* = 0.38) after age and sex adjustment. With additional adjustment for education and cardiometabolic risk factors, the results were largely similar (Table S11). Among GS older adults with DNAm GDF15 data (*n* = 7690; *M* age: 60.5 [7.2 SD]; 58.0% female; median time‐to‐event = 9.17 years; 208 dementia cases), DNAm GDF15 was unrelated to dementia risk (HR: 1.07; 95% CI: 0.91–1.25; *p* = 0.42) after age and sex adjustment. However, a one SD increase in plasma GDF15 was associated with an 11% increase [HR: 1.11; 95% CI: 1.02–1.22] in dementia risk after adjusting for age and sex (*p* = 0.02). With additional adjustment for education and cardiometabolic risk factors, the results were attenuated for plasma GDF15 [HR: 1.08; 95% CI: 0.97–1.21] (Table S11). Results accounting for relatedness (Cox mixed effects models) in Generation Scotland are presented in Table S12.

We next used data from the ARIC study to determine whether midlife DNAm CRP and GDF15 measures were associated with 25‐year dementia risk (*n* = 2606; *M* age: 57.0 [5.7 SD]; 42.0% female; median follow‐up = 20.2 years; IQR = 14.8–26.6 years; 585 dementia cases). A one standard deviation increase in DNAm CRP was associated with a 19% increase [HR: 1.19; 95% CI: 1.08–1.31] in dementia risk after adjusting for age and sex (*p* = 0.0003; Figure 5). By comparison, plasma CRP was not associated with dementia risk (HR: 0.96; 95% CI: 0.88–1.04, *p* = 0.35) after age and sex adjustment. With additional adjustment for education and cardiometabolic risk factors, the results were largely similar for DNAm CRP [HR: 1.11; 95% CI: 1.009–1.22] and plasma CRP [HR: 0.88; 95% CI: 0.80–0.96] (Table S11). A one standard deviation increase in DNAm GDF15 and plasma GDF15 was associated with a 16% [HR: 1.16; 95% CI: 1.07–1.27, *p* = 0.0004] and 27% [HR: 1.27; 95% CI: 1.16–1.39, *p* = 2.5E07] increase in dementia risk, respectively, after age and sex adjustment. With additional adjustment for education and cardiometabolic risk factors, the results were largely similar (Table S11).

## 4 Discussion

The present study used an epigenetic and proteomic approach to characterize two measures of chronic inflammation—CRP and GDF15 DNAm—and examine their associations with structural brain and cognitive trajectories, as well as dementia risk. Our primary findings illustrate that higher DNAm CRP and DNAm GDF15 scores—indicative of greater chronic inflammation—were associated with steeper declines in brain volume and cognition. Leveraging two external cohorts, we subsequently found DNAm CRP was associated with 18‐ and 25‐year dementia risk, whereas the results were mixed for DNAm GDF15. Compared to plasma levels of CRP, DNAm CRP appeared to show a stronger association with brain atrophy, cognitive decline, and dementia risk. Relative to plasma GDF15, DNAm GDF15 appeared to be more strongly associated with cognitive trajectories; however, this finding did not extend to brain volume trajectories or dementia risk.

Generally, CRP is a hepatic protein supporting the release of pro‐inflammatory cytokines (Muresan and Slevin 2024). Previous work has suggested that DNAm measures of CRP capture long‐term inflammatory burden (Conole et al. 2021; Hillary et al. 2024). In a proteome‐wide analysis, we found that a greater CRP DNAm score was associated with activated pro‐inflammatory pathways, including IL‐6/JAK/STAT3, type I interferon, PPAR, PD‐1, and complement signaling. Previously, DNAm CRP has been found to outperform plasma CRP in the case of cross‐sectionally measured neurocognitive outcomes, including cognition, total brain volume, normal‐appearing white matter, and gray matter volume (Conole et al. 2021). Here, we report comparable results for DNAm CRP and highlight that an elevated DNAm CRP score is also consistently associated with subsequent cognitive decline and brain volume loss. Overall, our results support DNAm CRP as a marker of chronic inflammation that is strongly associated with brain neurodegeneration, cognitive decline, and dementia risk.

GDF15, a cytokine and a member of the transforming growth factor‐β super family, has been identified as an important immunomodulatory factor (Isik et al. 2024). Our proteome‐wide analyses indicated that a greater GDF15 DNAm score reflects activated ephrin receptor signaling, extracellular matrix organization, TNF receptor activity, neutrophil degranulation, and IL‐6/JAK/STAT3 signaling. Previously, GDF15 has been consistently reported as being upregulated in response to neurodegeneration (Isik et al. 2024) and associated with elevated risk for several other age‐related diseases (e.g., coronary artery disease, myocardial infarction, cardiometabolic stroke) (Gadd et al. 2024; Isik et al. 2024). Like DNAm CRP, DNAm GDF15 is one of seven proteins included in the GrimAge (version 2) calculation, a proxy related to biological age and age acceleration (Lu et al. 2019). GrimAge has been previously found to be associated with white matter degeneration (Newman et al. 2022), morbidity (Lu et al. 2022), brain volume (Hillary et al. 2021), and cognitive decline (Hillary et al. 2021). On its own, elevated DNAm GDF15 has also been associated with increased risk of incident dementia, type 2 diabetes, and ischemic stroke (Gadd et al. 2024). The present findings extend previous work by demonstrating that a DNAm GDF15 score is associated with accelerated brain volume loss over time, including in regions vulnerable to atrophy (e.g., SPARE‐AD) in the context of neurodegenerative disease. Despite an overlap of only 12%–36% of variation between DNAm GDF15 and plasma GDF15 across the three cohorts, the two measures showed relatively similar associations with dementia risk and ADRD‐related brain atrophy; however, we found the former was a stronger predictor of cognitive decline.

Although the current study has notable strengths, such as the modeling of 3T MRI and cognitive trajectories over eight visits (up to 22.5 years) in a large longitudinal sample and a validation of the DNAm‐dementia associations in two separate cohorts, there are several limitations that should be considered. First, for the plasma comparison in BLSA, only 186 participants had concurrent visits for plasma and epigenetic measures. For individuals with non‐concurrent visits, we selected the closest plasma visit possible—which occurred either before or after the visit with the epigenetic measurement. For individuals with no epigenetic data, we selected the first available plasma visit in BLSA. As such, the mean follow‐up time in the plasma samples is somewhat longer compared to the epigenetic samples (Table S1). Second, because the BLSA—our primary cohort—is a primarily well‐resourced and highly educated sample, the results may not be directly generalizable to other populations. However, the validation with the ARIC cohort shows that our findings may be generalizable to more diverse populations. Third, we only considered DNAm measures at one time point. The investigation of longitudinal changes in DNAm measures to further test stability and prediction ability across time is warranted in future studies. Lastly, we acknowledge that the DNAm measures of CRP and GDF15 are not fully specific to inflammation and are influenced by various factors (e.g., genomic instability) associated with inflammation. However, we view these measures as downstream indicators of inflammation that remain relevant for brain and cognitive aging outcomes. Despite these limitations, the current study demonstrates that epigenetic proxies of CRP and GDF15 exposure reflect the activation of immune/inflammation‐related pathways associated with accelerated brain atrophy, cognitive decline, and long‐term dementia risk.

## Author Contributions

S.M.D. and K.A.W. conceived the study and developed the statistical analysis plan. S.M.D., J.C., C.B., M.R.D., D.C., and H.M.S. conducted statistical analyses for this manuscript. S.M.R., L.F., K.A.W., R.E.M., S.S., C.D., R.F.G., P.K., and A.Z.M. contributed data and resources. S.M.D. prepared the first draft of the manuscript. All authors contributed to the revision of the manuscript and reviewed and approved the final manuscript.

## Conflicts of Interest

R.E.M. is an advisor to the Epigenetic Clock Development Foundation and Optima Partners Ltd.

## Supporting information


**Data S1:** acel70281‐sup‐0001‐DataS1.xlsx.

## Data Availability

All data generated in the present study are included in this article, available on reasonable request, or in an online public repository. BLSA participants did not consent to unrestricted data sharing at the time of the study conducted. Researchers are welcome and encouraged to request use of BLSA data for scientific projects. Anonymized data not published within this article may be shared upon request from qualified investigators for purposes of replicating procedures and findings. Researchers who wish to use BLSA data are encouraged to develop a pre‐analysis plan that can be submitted for approval (https://blsa.nih.gov/blsa‐data‐use). BLSA proteomic data are publicly available via the Alzheimer's Disease Data Initiative as part of the participation in the Global Neurodegeneration Proteomics Consortium (https://www.neuroproteome.org). To apply and request access to BLSA proteomics data for purposes of reproducibility, researchers should submit a pre‐analysis plan for approval (https://blsa.nih.gov/blsa‐data‐use; https://blsa.nia.nih.gov/how‐apply). All components of GS received ethical approval from the NHS Tayside Committee on Medical Research Ethics (REC reference no. 05/S1401/89). GS has also been granted Research Tissue Bank status by the East of Scotland Research Ethics Service (REC reference no. 20‐ES‐0021), providing generic ethical approval for a wide range of uses within medical research. All participants signed a broad consent form. According to the terms of consent for GS participants, access to data must be reviewed by the GS access committee. Applications should be sent to access@generationscotland.org.
